# Mindfulness Meditation and Fantasy Relaxation in a Group Setting Leads to a Diminished Sense of Self and an Increased Present Orientation

**DOI:** 10.3390/bs9080087

**Published:** 2019-08-15

**Authors:** Niko Kohls, Tobias Esch, Lea Gerber, Lucas Adrian, Marc Wittmann

**Affiliations:** 1 Division of Integrative Health Promotion, Department of Social Work and Health, University of Applied Sciences Coburg, D-96450 Coburg, Germany; 2 School of Medicine, Faculty of Health, Witten/Herdecke University, D-58448 Witten, Germany; 3 Department of Psychology, University of Freiburg, D-79085 Freiburg, Germany; 4 Institute for Frontier Areas of Psychology and Mental Health, D-79098 Freiburg, Germany

**Keywords:** mindfulness meditation, fantasy relaxation, time perception, self-awareness

## Abstract

(1) Background: Mind-body interventions (MBI), such as meditation or other relaxation techniques, have become the focus of attention in the clinical and health sciences. Differences in the effects of induction techniques are being increasingly investigated. (2) Methods: Here, we compared changes in the individual experience of time, space, and self in 44 students in an integrative health-promotion program. They participated in a study employing mindfulness meditation and a relaxation intervention with one week between sessions, thus employing a within-subjects design. (3) Results: No significant differences were detected when subjective reports were compared directly after each intervention. However, we found significant sequence effects between t1 and t2, independent of the meditation type. The sense of self diminished, the present orientation increased, and the past and future orientations decreased in both interventions. (4) Conclusions: We propose using scales to assess subjective time, self, and space as basic constituents of experience to measure the specificity of intervention methods, as well as longitudinal changes.

## 1. Introduction

In the last decades, mindfulness-meditation techniques (MMT), as well as mind-body interventions (MBI), have become a focus of attention in the clinical and health sciences [1]. MMTs and MBIs focus on the relationships among the brain, mind, body, and behavior, and their effect on health and disease [2]. For example, a 2018 systematic review of 24 Randomized Controlled Trials (RCT) studies demonstrated that MBIs have small-to-moderate positive effects on heart failure patients’ objective and subjective outcomes [3]. MBIs were also found to be able to alter the expression of our genes, alter chromosomal telomere lengths, or mitochondrial metabolism, and also potentially reduce risk for certain disease [4,5]. A 2017 review showed that MBIs can reverse certain molecular reactions, essentially generating the “opposite of the effects of chronic stress on gene expression,” which could lead to a reduced risk of inflammation-related diseases [6]. Results from a 2016 study that compared popular MBIs led researchers to conclude “mind-body interventions can improve a person’s level of mental health when compared to those who do not practice these techniques” [7]. 

A substantial amount of research has also investigated the long-term effects of MMTs and MBIs on basic psychological functions and processes, such as executive functions [8], attention regulation, cognitive flexibility [9], bistable imagery [10], and time perception [11,12,13,14,15,16]. An interesting research branch has begun focusing on the short-term effects of meditation MMTs and MBISs and underlying trait characteristics. For example, research studies have shown that a four-to-five-day meditation training unit can enhance the ability to sustain attention in a way that was previously only observed in long-term meditators [17,18]. The aim of the present study was to investigate the transitory effects on experienced states of consciousness and what changes functionally in psychological variables associated with self, space, and time perception during meditative states [19]. 

An interesting question relates to whether, when, and how different MBIs exert a specific effect on psychological functions and whether the observed effects are generic and independent of the technique employed, e.g., related to general relaxation. From the taxonomy of MBIs, it can be concluded that some techniques may influence different psychological mechanisms associated with different degrees of introspective and interoceptive processes pertaining to body schema, proprioception, and to imagination and visualization [20]. A well-known technique employing body schemata and proprioception is mindfulness breathing meditation focusing on the embodied present moment experience, while guided imagery is more associated with visualization and mentalization.

The techniques associated with mindfulness meditation are probably the most frequently studied. As part of this method, the “body-scan” focuses on the perception of present-moment experience associated with the current mind-body status [21]. The specific aim of the “body scan” is to focus attention on successive parts of the body, frequently beginning with a mental scan of the left foot and ending with the top of the head, to become more mindfully aware of the precise bodily feelings and sensations [22]. Alternatively, individuals focus on breathing in and out. In fantasy-guided imagery, the mind is directed to intentionally visualize places, objects, or events that are not externally present with the aim of influencing psychological and physiological states [23]. Thus, given the different focus of attention in the two interventions one could assume that individuals engaging in mindfulness breathing meditation would experience different states of mind than subjects embarking in guided imagery with regard to their perception of time and space correspondingly also exhibiting impact upon their self-perception pattern. 

To empirically address the question of whether different short-term meditative and relaxing settings produce specific or generic-state changes directly after meditation, we conducted a study with individuals moderately familiar with meditation and relaxation techniques. We recruited volunteers in an undergraduate integrative-health-promotion program at the University of Applied Sciences Coburg (CUAS). We assessed and compared subjective time, space, and self as basic constituents of conscious experience during a meditation and fantasy-relaxation intervention session. The two induction methods are different in how attention is directed either to the body (meditation) or to an imagined world (fantasy-relaxation). Since the senses of self, time, and space have been shown to be modulated during meditative practice [13,15,24], we hypothesized that these aspects of conscious awareness should be more strongly affected after a meditation, as compared to fantasy relaxation.

## 2. Materials and Methods

### 2.1. Participants and Procedure

Forty-four second year bachelor students enrolled in the integrative health promotion program at CUAS (37 women, 7 men) aged between 18 and 35 years (mean age: 23.9 years; S.D. = 5.4) participated in this study. The students had already been familiarized with stress reduction, relaxation and meditation techniques as part of their training. Thus, they had some prior exposure to both types of relaxation inductions utilized in this study. They had been introduced to the theory of mind-body techniques and also participated in an 8-week mind-body related stress-reduction program in the foregoing semester, which has been described and evaluated elsewhere [25,26]. The current study was conducted at the end of the fourth semester, after the students had been introduced to different forms of relaxation techniques including mindfulness meditation, walking meditation, fantasy journey, as well as yoga, progressive muscle relaxation, and autogenic training under the supervision of two mind-body experts for the whole semester (NK and TE). They were randomly assigned to two different experimental groups (*n* = 22 in each class). The two groups received the same intervention only in a different temporal order: One group (*n* = 22) started with the mindfulness breathing meditation (t1), and the other (*n* = 22), with the guided fantasy relaxation (t1). The two groups switched interventions (t2) one week later. 

At the first session, before the respective intervention was conducted, participants in each group filled out the three trait questionnaires described below. Then, the guided intervention either mindfulness breathing meditation or fantasy relaxation lasting exactly 10 min was conducted, after which the students filled out the state questionnaire pertaining to their experiences during the intervention. The audio scripts of both interventions are presented in the Appendix A both in the German original version as well as the English language translation. One week later, the same formal set up for the alternate intervention was used and after the intervention, the state questionnaire was again administered. 

All students were informed about the study purpose and asked whether they were willing to take part during a lesson conducted a week before the study without financial compensation. After the second session, students were given the opportunity to further inquire about the aim and hypotheses of the study as well as the theoretical framework. Participation throughout the study was voluntary. Our study was not linked to any kind of examination students had to take to pass the seminar and the experiment took place after the exams had been conducted. The scientific and value-oriented principles as defined and described by CUAS, served as the fundamental ethical frame for our study, where the combined first-hand-experience of research with lecturing in a classroom setting is considered to be a fundamental educational pillar. The project was categorized as an internal review board (IRB) exempt project and according to the ethical guidelines [27] an IRB waiver was obtained and the study number CUAS-SW-NK-002 assigned. The study was conducted according to the ethical principles of the Declaration of Helsinki.

### 2.2. Interventions

Both mindfulness meditation and fantasy relaxation were recorded interventions in the German language that lasted ten minutes and were guided by the same audio-taped female voice (Lea Gerber).

An excerpt from a recorded, commercially available body-scan meditation was used for the meditation intervention [28] for a transcript, as seen in Appendix A. In the meditation recording, participants are asked to first pay attention to their breath and subsequently to several body parts. We transcribed the relevant text passages and had it spoken by co-author Lea Gerber.

The fantasy journey was produced especially for this study (by Lea Gerber and Lucas Adrian) because there were no commercial products available, which fit our needs. We thereby, achieved comparability with the meditation recording in tone, voice modulation, wording, and length. In the recording, an island scene is described with various animals and ships appearing on the scene, but without any real narrative (for a transcript see Appendix A).

### 2.3. Trait Inventories

#### 2.3.1. Barratt Impulsiveness Scale (BIS-11) 

The Barratt Impulsiveness Scale (BIS) consists of 30 items ranging from 1 (very untrue) to 5 (very true) which are grouped into three subscales: non-planning impulsivity, motor impulsivity, and attention/cognition impulsivity [29]. Sample items include “I plan trips well ahead of time.” and “I spend or charge more than I earn.” According to the validated German version, it is recommended to rely more on the sum score than on individual subscales [30].

#### 2.3.2. Zimbardo Time Perspective Inventory (ZTPI) 

The validated German version of the ZTPI has 56 items ranging from 1 (rarely/never) to 4 (almost always) which are grouped into five subscales representing orientation towards the following dimensions: past-negative (“I often think about the bad things that have happened to me in the past.”); past-positive (“Happy memories of good times spring readily to mind.”); present-hedonistic (“I take risks to put excitement in my life.”); present-fatalistic (“Because things always change, one cannot foresee the future.”); and future (“I am able to resist temptations when I know that there is work to be done.”) [31,32]. 

#### 2.3.3. Freiburg Mindfulness Inventory (FMI) 

The FMI contains 14 four-point items with answer categories ranging from 1 (rarely) to 4 (almost always) which evaluate mindfulness on the basis of a two-factor structure [33,34,35]. The two factors are “presence” as ability to attend to the present moment (“I am open to the experience of the present moment”) and “acceptance” as non-judgmental attitude (“I am patient with myself when things go wrong”) [36]. The factor presence is of special interest as it is conceptually discussed as the propensity to attending mindfully to the “here and now”.

### 2.4. State Inventory

#### Inventory on Subjective Time, Self, Space (STSS)

The STSS has previously been used with varying instructions to assess states of consciousness during a real waiting time [37], while watching two different dance performances [38] and during a depth-relaxation meditation [19]. Participants had to fill out five different visual-analogue scales (VAS). (1) The intensity of awareness of the bodily self and (2) space were assessed with two non-verbal pictorial scales containing answer categories ranging from 0 to 6. The questions were: “How intensively did you experience yourself?” and “How intensively did you experience the surrounding space?” Higher scores indicate greater awareness of body and space. (3) 100-mm-line VASs were presented with the following questions: (3) “How intensively did you think about time?” (anchor points: not at all—extremely); (4) “How fast did time pass for you?” (extremely slowly—extremely fast). (5) Finally, a 100-mm line had to be subdivided into three parts (making two marks) representing the degree of orientation towards the past, present, and future during the intervention.

### 2.5. Statistical Analyses

Within-subject differences for the type of intervention (mindfulness meditation, fantasy relaxation), as well as the session number (t1, t2), were assessed using two-sided t tests for the measures of subjective time, self, and space (STSS). Pearson correlations for associations between the three trait questionnaires and the state scale (STSS) were additionally calculated. The false-discovery-rate (FDR) method, a multiple-comparison-correction procedure [39], controls for multiple tests with an initial *p* value set to 0.05. For statistical calculations we used SYSTAT 13 for Windows.

## 3. Results

In comparing the two intervention conditions (meditation, fantasy-relaxation) over the two time points (t1, t2) we performed separate ANOVAs for the seven state variables. In our case we have two groups of participants who either started with meditation and then did the fantasy-relaxation condition (group 1), or performed the interventions the other way around (group 2). According to this logic the main factor ‘group’ should not lead to differences in the dependent variables as it tests for the between-subjects factor group. Subjects in both groups performed in both conditions and thus there should not be an overall effect for the dependent variables. The group × condition interaction reflects the within-subjects’ differences of the two groups starting with a different intervention condition and thus reflecting differences between t1 and t2 (a potential learning effect over time).

The main factor group did not show a significant effect in any of the seven dependent variables (all F < 1; all *p* > 0.1). The groups starting either with meditation or with relaxation scored equally in the questionnaires assessing the two conditions. One significant effect of the main factor condition appeared for the variable ‘Intensity of the sense of self’ (F = 8.1; *p* < 0.007). The interaction group × condition, reflecting differences between t1 and t2 as a learning effect over time, showed four significant calculations, namely for the dependent variable of ‘Intensity of the sense of self’ (F = 10.5; *p* < 0.002), % Sense of past (F = 5.8; *p* < 0.021), % Sense of present (F = 23.7; *p* < 0.001), and % Sense of future (F = 23.1; *p* < 0.001).

For a better understanding of the above effects revealed by the ANOVAs, in Table 1 we present a table of descriptive mean values in the two conditions and corresponding *t* tests for the answers of the conscious-state scale (STSS) filled out after the mindfulness meditation and the fantasy relaxation. Only one difference proved to be marginally significant, reflecting the main effect of condition in the above ANOVA. The sense of self after fantasy relaxation (3.30) was higher than after mindfulness meditation (2.64) (t = −2.6, p = 0.014; not significant after FDR for 7 calculations). 

According to the ANOVA there were several interaction effects of group × condition, reflecting a sequence effect t1 to t2. Each participant took part in both sessions (with one week between sessions). Therefore, in Table 2 we present the analysis of a potential learning effect. According to t tests, four of the seven variables showed a significant change in average responses. First of all, the sense of self diminished between t1 (3.34) and t2 (2.59) (t = 3.0, *p* = 0.004). All three dimensions of time orientation changed. The present orientation increased at t2 (61.3) vs. t1 (42.9) (t = −4.9, *p* < 0.001); accordingly, past orientation decreased at t2 (17.6) as compared to t1 (22.6) (t = 2.4, *p* = 0.019); a similar decrease was seen for the future orientation (t2: 20.8; t1: 34.5) (t = 3.0, *p* < 0.001). 

Which individual trait correlated with the four state changes in meditation/relaxation over time? The question, which personal disposition enhanced the state effects of our interventions can be obtained from Table 3. For example, mindfulness as a trait could have been sensitive for state effects, but correlation coefficients were not related. Two related trait variables were significantly associated: impulsivity and present hedonism. The more impulsive individuals were, the less increase in the present orientation was experienced over the two sessions (r = −0.473, *p* = 0.001), and the more future oriented they were (r = 0.429, *p* = 0.004). Trait impulsivity counters the effects of meditation/relaxation. Similarly, the more present hedonistic individuals were, the less increase in present orientation they experienced (r = −0.420, *p* = 0.004), and a stronger future orientation was observed (r = −0.473, *p* = 0.002).

## 4. Discussions

In this study, we compared the changes observed in the experience of time, space, and self in students in an integrative health-promotion program taking part in both a mindfulness meditation and a relaxation intervention, employing a within-subject design. The main aim of our study was to compare the effects of two different relaxation techniques, mindfulness meditation and fantasy relaxation, in a group of students with basic experience with those induction methods.

The results indicate that there is one significant difference between the two types of interventions, namely the intensity of the self was observed to be stronger after the guided imagery intervention (significant after the ANOVA). Several significant effects were found when comparing the two intervention time points, regardless of intervention type (ANOVA interaction group × condition). For a better understanding of effects, we collapsed the questionnaire answers of 44 subjects at t1 and t2 regardless of the underlying intervention and thus treated the interventions as equal (as indicated by the null results for differences between intervention types). Effects for a sequence effect were found in four of the seven variables: the sense of self, as well as the past and future time orientation, decreased, while presence orientation increased. Individuals had stronger experiences that are typically found in meditative states at t2 than at t1. The decrease of the sense of self, as well as a stronger sense of presence (at the expense of the past and future orientation) is a typically sign of altered states of consciousness in different relaxation techniques [13,15,24]. Individuals get more absorbed in the ‘here and now’ and show less rumination towards past and future events. The state of absorption in the object of meditation and relaxation is also accompanied by a decrease of the sense of self, a sort of self-transcendence.

Effects on long-term stable traits in experienced meditators show a decrease in rumination and mind-wandering, as well as an increase in self-regulation [17,40,41,42]. Transitory meditative-state effects can similarly be described as an increased sense of presence at the expense of momentary rumination in the past and future. Transitory effects in more experienced meditators have also been described as losing of the sense of self and time, effects which are achieved through an increased presence orientation [11,12,15,43]. Our ‘learning’ effect visible as differences between t2 and t1 corresponds with this concept, as an increased present state at the expense of the past and future awareness accompanies a decrease in the awareness of the self. The relaxation intervention and the mindfulness meditation both led to these changes. Past and future ruminations were suppressed while momentarily focusing on the induction of imaginary images. The immersive experience of listening to the imaginative story apparently led to a loss of the sense of self. 

First-order correlations were detected between the variables that were sensitive to experience changes during the intervention and the three trait-questionnaire instruments tapping into mindfulness, impulsivity, and time perspective. There were moderate associations between the two trait variables hedonistic present orientation (ZTPI) and impulsivity (BIS) and the state variables of present and future orientation during meditation. The more impulsive and hedonistic present-oriented the students were as a personality trait, the less presence orientation they experienced during meditation and the more short-term future oriented they were. Short-term future orientation as a state variable should not be confounded with the long-term future orientation as a trait. More impulsive individuals typically are less future-orientated as a general trait, i.e., they tend to make fewer plans for the future as they concentrate on savoring the present, or the very-near future [31]. Future orientation as a state variable in our context refers to the inability to get absorbed in the present-moment relaxation or meditation inductions. Rather, individuals who are more impulsive or present-hedonists are less able to stay in the present moment and instead focus their attention on the near future, the end of the 10 min intervention.

Moreover, to understand these outcomes, one has to differentiate between an impulsive and hedonistic present orientation and present-mindedness as trained in meditation techniques. The former is associated with a strong urge to act at the present moment, whereas the latter is associated with an observational state associated with more self-control [44]. Our results clearly show that individuals who are more impulsively and hedonistically present oriented have a weaker present orientation and a stronger short-term future orientation while meditating. They have less ability to immerse themselves in meditative or imaginative present awareness.

The fact that we did not identify setting-dependent differences between mindfulness meditation and guided imagery after a 10 min intervention in our study suggests that both interventions—at least if applied for this period of time—produce similar effects that have to be considered generic. The fact that meditation and guided imagery showed no differences with reference to the primary research question in our study could be a result of their common relaxation physiology, i.e., relaxation response pathways [45,46]. The relaxation response (RR) is defined by a set of integrated physiological mechanisms and ‘adjustments’ that are elicited when a subject engages in a repetitive or focused mental or physical activity and passively ignores distracting thoughts. Such behaviors seen in meditation, certain forms of prayer, tai-chi/qigong, yoga, autogenic training, and visualization and guided-imagery procedures, etc., are associated with instantly occurring physiological changes that include decreased oxygen consumption or carbon dioxide elimination (i.e., reduced metabolism), and a lowered heart rate, arterial blood pressure, and respiratory rate [47]. These innate processes might also correspond with an altered state of self or self-perception.

Alternatively, time exposure in the specific setting might have been too short to produce specific effects. Given that we found slightly higher scores in intensity of the self in the guided-imagery class, we believe that a longer intervention period might have produced more pronounced effects. We suggest continuing investigations of state-dependent differences attributable to different MBI settings [48,49] to assess the specificity of intervention mechanisms. A general understanding of different induction methods for altering states of mind could enable us to specifically apply meditation and relaxation methods for different clinical purposes and groups [40,41,50]. The core features of altered states of consciousness are antithetical to psychiatric symptoms [51]. They lead to less awareness of the self and time. In many psychiatric syndromes, such as anxiety and depression, individuals show hyper-awareness of the self and of time. If a person is hyper self-aware, the negative affect is high, and time drags. That is why meditation might have positive effects as intervention in such a health-related context [7,25,26,49].

## Figures and Tables

**Table 1:** Mean (S.D.) values for the conscious-state inventory on subjective time, self, and space (STSS) for the mindfulness meditation and the fantasy-relaxation intervention.

Measure STSS	Mindfulness Meditation Mean (S.D.)	Fantasy Relaxation Mean (S.D.)
Intensity sense of self [0 … 6]	2.64 * (1.1)	3.30 * (1.5)
Intensity sense of space [0 … 6]	4.25 (1.3)	4.09 (1.7)
Intensity sense of time [0 … 100]	33.6 (26.1)	40.2 (24.0)
Speed of time passage [0 … 100]	58.8 (23.6)	58.1 (18.1)
% Sense of past [0 … 100]	20.6 (13.1)	19.6 (13.3)
% Sense of present [0 … 100]	51.3 (22.3)	52.9 (23.7)
% Sense of future [0 … 100]	27.8 (15.5)	27.5 (18.7)

* *p* < 0.05.

**Table 2:** Mean (S.D.) values for the conscious state inventory on subjective time, self, space (STSS) for the two sessions (t1, t2), regardless of whether mindfulness meditation or the fantasy relaxation was conducted.

Measure STSS	Session t1 Mean (S.D.)	Session t2 Mean (S.D.)
Intensity sense of self [0 … 6]	3.34 (1.2) **	2.59 (1.4) **
Intensity sense of space [0 … 6]	3.95 (1.5)	4.39 (1.5)
Intensity sense of time [0 … 100]	38.0 (2.5)	35.8 (2.6)
Speed of time passage [0 … 100]	58.3 (22.9)	58.6 (19.0)
% Sense of past [0 … 100]	22.6 (14.6) **	17.6 (11.0) **
% Sense of present [0 … 100]	42.9 (24.0) **	61.3 (17.7) **
% Sense of future [0 … 100]	34.5 (19.0) **	20.8 (11.6) **

** *p* < 0.05 significant after FDR adjustment.

**Table 3:** Pearson correlation coefficients among the variables of the STSS that were sensitive to changes between t1 and t2 and the personality scales of impulsivity (BIS), time perspective (ZTPI), and mindfulness (FMI).

	Intensity Sense of Self [0 … 6]	% Sense of Past [0 … 100]	% Sense of Present [0 … 100]	% Sense of Future [0 … 100]
BIS Sum	−0.057	0.260	−0.473 **	0.429 **
ZTPI present hedonistic	0.026	0.153	−0.420 **	0.447 **
ZTPI present fatalistic	0.115	0.272	−0.241	0.116
ZTPI future	−0.189	−0.180	0.245	−0.179
ZTPI past negative	−0.211	−0.010	0.214	−0.288
ZTPI past positive	−0.073	−0.087	−0.224	0.368 *
FMI presence	0.002	−0.164	0.176	−0.095
FMI acceptance	0.030	−0.165	−0.023	0.175

* *p* < 0.05, ** significant after FDR-adjustment.

## Appendix A

### Appendix A.1. Transcript of the Mindfulness Meditation (Original)

Nehmen Sie eine Haltung ein, die sich für Sie im Moment am passendsten anfühlt und lassen Sie sich ein auf die Wahrnehmung des Atems, wie er in Ihrem Körper ein und ausströmt, während Sie hier sitzen oder liegen. Gehen Sie in Kontakt mit den Empfindungen des Atmens, überall dort, wo diese am lebendigsten für Sie sind. Vielleicht an den Nasenflügeln oder im Bauchbereich. Oder auch im Brustraum und reiten Sie Moment für Moment auf den Wellen Ihres Atems. Erleben Sie die gesamte Dauer des Einatmens und die Zeit die der Atem braucht, um den Körper wieder zu verlassen. Richten Sie die Aufmerksamkeit auf diese Atemempfindungen und halten Sie sie dort, so gut Sie können. Verweilen Sie in diesem Gewahrsein des Atems, Augenblick, für Augenblick und Atemzug für Atemzug. Und wenn Sie möchten können Sie nun den Fokus Ihrer Aufmerksamkeit auf den Kontakt des Körpers mit dem Stuhl oder dem Boden richten und die damit verbundenen Empfindungen spüren.

Erlauben Sie diesen Empfindungen genauso zu sein, wie sie sind. Ganz egal, ob Sie sie eher als angenehm oder unangenehm oder neutral empfinden. Bleiben Sie einfach bewusst bei diesen Empfindungen in einem Gewahrsein, das gleichzeitig auch den Atem umfasst bemerken Sie einfach, wie der Atem alle Empfindungen von Kontakt, von Berührung von Temperatur und Druck, Härte und Weichheit mit umfassen kann wie der Atem diese Empfindungen gleichsam in Aufmerksamkeit badet. Nehmen Sie die Empfindungen genauso wahr, wie sie sind. Außerhalb des Denkens, nur als reine Empfindungen, ob sie schwach sind, mittel, oder stark in ihrer Intensität, ob angenehm, unangenehm oder neutral, Sie brauchen nichts an ihnen zu verändern. Öffnen Sie sich ihnen einfach nur mit Bewusstheit während Sie im gegenwärtigen Moment ruhen, so wie er ist, Augenblick für Augenblick für Augenblick. Und wenn Sie bereit sind, erlauben Sie sich jetzt das Feld ihres Gewahrseins noch weiter auszudehnen, so dass es die ganze Haut und die Luft um den Körper herum miteinschließt und die Wahrnehmung des Körpers als Ganzes wie er hier sitzt oder liegt und atmet. Sodass Ihr Gewahrsein dieses ganze Universum von Empfindungen einschließt, das man den Körper nennt oder das man als die Erfahrungslandschaft des Körpers bezeichnen könnte vielleicht nehmen sie weiterhin auch noch die Empfindungen des Atems wahr, der ein und ausströmt aber jetzt im größeren Kontext des gesamten Körpers, der atmend hier sitzt oder liegt. Während Sie einfach nur hier verweilen, im Bewusstsein des Körpers als Ganzes in seiner Vollständigkeit. In seinem ganz eigenen Dasein. Umfasst Ihr Gewahrsein den Körper. Erfüllt den Körper. Umgibt ihn. Fließt durch ihn hindurch, überall. So wie auch der Atem überall hinfließt. Vielleicht können Sie sogar spüren oder sich vorstellen, wie Ihre Haut atmet, was sie in der Tat auch tut.

English translation

Assume a comfortable position and let yourself experience the sensation of the breath flowing in and out of your body. Get into contact with the sensation of breath everywhere it feels most lively for you—maybe in your nostrils, or belly, or chest—and ride, moment for moment, on the waves of your breath. Experience the whole duration of breathing in and the time your breath needs to leave your body again. Direct your attention to these sensations of breathing and keep them as best you can. Rest in this awareness of your breath, moment for moment and breath after breath. If you want, you can now direct the focus of your attention to your body’s contact with the chair or the floor and feel these sensations. Allow them to be exactly the way they are, whether you find them pleasant or unpleasant or neutral. Just stay consciously with these sensations in an awareness that simultaneously includes the breath. Simply notice how your breath can enclose all the sensations of contact, touch, temperature, pressure, hardness, and softness. As if your breath were to bathe these sensations in awareness. Perceive these sensations just as they are, outside of thought, only as pure sensation, whether they are weak, average, or strong in their intensity. Whether they are pleasant, unpleasant, or neutral; you do not need to change anything about them. Just open up to them consciously while you rest in the present moment, just as it is, moment for moment for moment. When you are ready, allow yourself to expand the realm of your awareness even further so that it includes your skin and the air around your body, as well as the perception of your body as a whole as it sits or lies here and breathes Your awareness contains this whole universe of sensations that one calls the body or that one could also call the landscape of experience of the body. Maybe you are still perceiving the sensations of the breath, flowing in and out, but now within the larger context of your whole body that sits or lies here breathing. While you do nothing but rest here, in awareness of the body as a whole, in its completeness, in its thoroughly own existence, your awareness encloses your body, fills it, surrounds it, flows through it, everywhere. Just like your breath is also flowing everywhere. Maybe you can even feel or imagine how your skin breathes, which, in fact, it does.

### Appendix A.2. Transcript of the Fantasy Relaxation (Original)

Stell dir eine Insel vor. Es ist eine Insel mit großen Bergen und weißen Sandstränden die immer wieder durch lange Steilküsten unterbrochen werden. Die Insel ist von türkisblauem Wasser umgeben. Das Wasser schimmert von den Strahlen der aufgehenden Sonne durchflutet und im flachen klaren Wasser tummeln sich Fische in allerlei Farben und Formen. In der Ferne sprüht ein Wal eine Wasserfontaine in die Luft und kurz darauf erscheint seine Flosse aus dem Wasser, verschwindet wieder taucht wieder auf, in einem regelmäßigen Rhythmus. Weiter hinten am Horizont erscheint ein großes altes Segelschiff mit weißen Segeln gleitet langsam dahin. Der Strand ist aus feinem Sand und überall liegen Muscheln, Algen und kleine Hölzer. Aus einer der Muscheln schaut vorsichtig ein kleiner Krebs heraus und beginnt den Strand entlang zu laufen. Über dem Meer kreisen Möwen und halten Ausschau nach dem ersten Leckerbissen des heutigen Tages, sobald sie einen erblickt haben, schießen sie im Sturzflug in Richtung Wasser, um dann mit einem Fisch im Schnabel wieder emporzusteigen. In den nahegelegenen Bäumen sitzen Affen. Es ist Frühling und gerade haben viele der Affen Junge. Die kleinen Affen hüpfen bereits zu früher Morgenstunde in den Baumwipfeln umher. Gerade spielen zwei der kleinen Affen Fange und kreischen ausgelassen und voller Freude. Ein anderer Affe wird von seiner Mama ausgiebig gelaust und freundlich umarmt. Ein paar der Affen schlafen noch genüsslich in den Ästen und lassen sich durch den Tumult der Kleinen nicht stören. Es ist noch ein wenig frisch und in den Gräsern und Sträuchern sammelt sich der Tau. Kleine Vögel sitzen in den Sträuchern und trällern ihr erstes Morgenlied. In der Ferne ist ein Wald zu erkennen. Ein Ameisenbär hat sich aus der Dunkelheit des Waldes auf die grüne, nasse Wiese gewagt und schnüffelt ein wenig herum. Beim Gekreische der Affen hebt er immer wieder kurz seinen Kopf, lässt sich dann aber nicht weiter davon stören und läuft weiter am Waldrand entlang, die Gegend erkundend. An den rauen Steilküsten der Insel bewegen sich die Grasbüschel im Wind und die Wellen schlagen an die Felsen. Die Felsen im Wasser sind aus grauem Gestein und werden von schwarzen Miesmuscheln und grünen Flechten geschmückt. In der Tiefe sind rote Korallen und Seesterne zu erahnen. Auf den kleinen Felsvorsprüngen weiter oben und in sicherer Distanz zum Wasser, haben die Möwen ihre Nester gebaut. Gerade eben kommt eine Möwe herangeflogen und füttert ihren kleinen Nachwuchs. Dann ist sie auch schon wieder weg, um Nachschub für die hungrigen Mäuler zu holen. So herrscht ein reger Flugbetrieb rund um die Felsen. Weiter hinten, im Innern der Insel gibt es hohe Berge mit weißen Schneegipfeln. Das Schmelzwasser fließt zunächst in kleinen Rinnsalen den Berg hinunter, weiter unten werden sie zu Bächen, um sich dann schließlich im Tal in einen reißenden Strom zu verwandeln. In all den Jahrzehnten hat der Fluss sich seinen eigenen Weg geformt und fließt nun in sich windenden Kurven durch das Tal. Durch enge Felsschluchten, durch tiefen Urwald mit Lianen, die bis ins Wasser reichen und vorbei an Wiesen mit herrlich blühenden Blumen bis er an einer Stelle der Insel in das weite Meer mündet. Mittlerweile ist die Sonne schon höher gestiegen und es ist wärmer geworden. Der Ameisenbär hat sich wieder in die Kühle des Waldes zurückgezogen. Die Affen sind nun alle erwacht, lausen sich, toben herum, Essen Nüsse von den Bäumen oder erkunden die nahe Umgebung. Die beiden kleinen Äffchen, die vorher noch wild fange gespielt haben klammern sich nun an die Bäuche ihrer Mütter und lassen sich von ihnen herumtragen. Die Krebse suchen Schutz vor der aufsteigenden und immer wärmer werdenden Sonne und haben sich in ihre Muscheln zurückgezogen. Der Sand wird heiß. Die Stunden vergehen und die Sonne steigt höher und höher. Während der Mittagshitze ist es ruhig auf der Insel. Die Affen Suchen sich ein Schattenplätzchen unter den vielen Bäumen und dösen ein wenig. Nur einer der kleinen Affen will nicht zur Ruhe kommen und hüpft wild in den Baumkronen umher. Als es Nachmittag wird ist Flugunterricht bei den Steilküsten. Manche der kleinen Möwen wagen sich an den Rand ihres Nests, stehen dort zunächst auf wackelnden Beinen und schauen in die Tiefe zum Meer hinab. Dann hüpfen die ersten Küken vom Rand und flattern ihren ersten richtigen Flug über das Wasser. Mit ihren kleinen Flügeln und in Begleitung der Mütter fliegen sie kleine Runden und steuern dann wieder ihr sicheres Nest an. Sicher gelandet, erhole sie sich eine Weile, um dann den nächsten Flug zu wagen. Das ist ein lebendiges Treiben. Der Wind hat ein wenig zugenommen und so schlagen nun größere Wellen an die Felswände und es schäumt gewaltig. Die Möwen jedoch lassen sich hiervon nicht aus der Ruhe bringen, gehört dies doch zum Alltag auf einer solchen Insel im Meer. Es wird langsam Abend, das große alte Segelschiff mit den weißen Segeln ist längst am Horizont verschwunden, jetzt ist nur ein kleines Boot in der Bucht einer nahe gelegenen Insel zu sehen, wahrscheinlich ankert es dort die Nacht über. In der Abenddämmerung kommt der Ameisenbär noch mal aus dem Wald heraus, reckt seine Nase in die Luft und atmet vergnügt ein wenig die kühle Meeresluft des Abends ein. Der Krebs in seiner Muschel läuft flink am Strand entlang und erfreut sich am kühlen Sand. Die Affen machen es sich auf den Bäumen zum Schlafen bequem und auch bei den Nestern an den steilen Felsküsten wird es ruhig.

Alle schlafen. Langsam berührt die Sonne den Horizont und taucht das Meer in ein orange- rotes Licht. Ein paar Möwen gleiten noch durch den Abendhimmel und ihre schlanken Silhouetten heben sich schwarz vor dem roten Himmel ab. Es ist bereits kühler geworden. Hinter den Schneebedeckten Gipfeln der Berge steigt der Mond empor und taucht den Schnee trotz der aufkommenden Dunkelheit in ein fahles Licht. Auf der Wiese hört man noch die Grillen zirpen, während sich das Gras hin und wieder in einer lauen Abendbrise wiegt. Die ersten Sterne stehen am Himmel. Ein Tag geht vorüber.

(English translation)

Imagine an island, an island with high mountains and white beaches that are only interrupted by steep cliffs. The island is surrounded by turquoise-blue water. The water shimmers from the rays of the rising sun, and in the shallow, clear water fish of many shapes and colors flit about. In the distance a whale sprays a jet of water into the air, and shortly afterwards, his tailfin breaks the water, disappears, and surfaces again, in a steady rhythm. Farther away, from beyond the horizon, a large, ancient sailboat with white sails becomes visible and glides away. The beach is made of fine sand, and there are shells, seaweed, and small pieces of wood everywhere. A small crab carefully peeks out from one of the shells and begins to skuttle along the beach. Seagulls hover above the ocean and keep a lookout for the first treat of the day. As soon as they have spotted one, they dive down towards the water only to rise again with a fish in their beaks. Monkeys are sitting in the nearby trees. It is spring, and many of the monkeys have just had babies. Early in the morning the baby monkeys leap around in the treetops. Two little monkeys are playing tag and shrieking joyfully. Another monkey is being hugged by its mother. A couple of monkeys are still sleeping between the branches and aren’t bothered by the noise of the younger ones around them. It still is a little bit chilly, and dew gathers in the grasses and the bushes. Small birds sit in the bushes and sing their first morning songs. There is a forest in the distance. An ant bear has dared to leave the darkness of the forest and sniffs around in the wet, green grass. It looks up again and again when the little monkeys shriek, only to continue undisturbed with his business of exploring his surroundings. On the rough, steep cliffs of the island the grass bends in the wind, and the waves crash against the rocks. The rocks in the water are made of grey stone and are decorated with black mussels and green seaweed. One can barely see red corals and starfish in the depths of the water. Seagulls have their nests on the little rock ledges further up at a safe distance from the water. Just now a seagull flies up and feeds its offspring. It quickly flies away again to get more food for the hungry chicks. Lots of gulls are flying around the rocks. Further back on the island there are high mountains with white peaks. The meltwater first flows down the mountains in little brooks before they form small streams that channel into a torrential river, the river has formed its own path over the decades and is now flowing in writhing curves through the valley. Through narrow canyons, through deep jungle with lianas that reach all the way into the water, passing by meadows with marvelously blooming flowers until it flows into the sea. Meanwhile, the sun has risen higher, and it has become warmer. The ant bear has retreated into the fresh coolness of the forest. The monkeys are now all awake, pick each other’s lice, jump around, eat nuts from the trees, and explore the close environment. The two little baby monkeys that were playing tag before now cling to the bellies of their mothers and enjoy being carried around. The crabs, looking for cover from the rising sun that is becoming hotter and hotter, have retreated into their shells. The sand becomes hot. The hours pass and the sun rises higher and higher. During the heat of midday, the island is calm. The monkeys look for shadowy places and doze away. Only one of the little monkeys does not want to rest and enthusiastically jumps around in the treetops. When afternoon arrives, flying lessons begin around the steep cliffs. Some of the little seagulls dare to walk up to the edge of their nests, stand there on shakey legs, and look down into the sea below. Then, the first chicks hop from the edge and flutter on their first real flight over the water. Accompanied by their mothers, they fly a few small laps with their little wings before returning to their nests. Safely landed, they recover for a while only to dare to start the next flight. It’s a lively business. The wind has picked up a bit, and now bigger waves are crashing against the rocks. The seagulls do not let themselves be disturbed by this, as it is commonplace on such an island. Dusk slowly falls. The large, ancient sailboat with the white sails has long disappeared over the horizon. Now only a small boat is visible in the bay of a nearby island. It will probably anchor there overnight. The ant bear leaves the forest once more, stretches its nose upward into the air and inhales the fresh evening air. The crab in its shell swiftly skuttles down the beach and relishes the cool sand. The monkeys get comfortable in the trees for the night, and it also becomes quiet in the nests in the steep cliffs. Everything is asleep. Slowly the sun touches the horizon and bathes the ocean in an orange-red light. A few seagulls are still gliding through the evening air, and their slender, black silhouettes stand out in the red sky. It has already cooled down. The moon rises from behind the snow-covered mountaintops and makes the snow glow palely in spite of the approaching darkness. You can hear crickets chirping in the meadows, while the grass rocks to and fro in the gentle night wind. The first stars appear in the sky. A day has passed.
